# Morphological Characteristics, Mitochondrial Genome, and Evolutionary Insights Into a New Sea Squirt From the Beibu Gulf

**DOI:** 10.1002/ece3.70639

**Published:** 2025-01-01

**Authors:** Yichuan Zhang, Yuting Qin, Yueying Wu, Liping Liu, Wenguang Zhang, Ling Ding, Xiangpei Ya, Zhiting Wen, Kuaili Feng, Hong Wang, Yujun Wang

**Affiliations:** ^1^ Guangxi Key Laboratory of Beibu Gulf Marine Biodiversity Conservation, Pinglu Canal and Beibu Gulf Coastal Ecosystem Observation and Research Station of Guangxi, Ocean College Beibu Gulf University Qinzhou China; ^2^ Institute of Sericulture, Applied Technology R and D Center for Special Sericulture of Hebei Province Universities Institute of Sericulture, Chengde Medical University Chengde China

**Keywords:** mitochondrial genome, morphological characteristics, phylogenetic evolution, sea squirt

## Abstract

A new species of the genus Microcosmus was described in this study based on specimens collected from the coast of Xilian Town, Xuwen County, Zhanjiang, Guangdong Province, China. The morphological and molecular characteristics of this new species, *Microcosmus* sp. z YZ‐2024 (YZ‐2024), distinguish it from other sea squirts. Firstly, YZ‐2024 can be preliminarily distinguished by the following morphological features: (1) smooth surface of the tunica, without protuberances; (2) except for the attachment site, the tunica is orange‐red; (3) absence of neural ganglia, and the tunica does not contract abruptly when disturbed; (4) the heart is oval‐shaped. Most importantly, the mitochondrial genome characteristics of YZ‐2024 effectively and accurately distinguish it from other congeneric species, with a mitochondrial genome size of 14,520 bp (GenBank No. PP067884) and the proportions of bases A, T, G, and C comprising 26.83%, 47.16%, 16.91%, and 9.10%, respectively. Except for the gene COX1, the mitochondrial genome arrangement of YZ‐2024 is totally different from that of 22 other species in the class Ascidiacea. Evolutionary analysis has indicated that YZ‐2024 clusters with its congeneric species *Microcosmus sulcatus*. Interestingly, YZ‐2024 belongs to Pyuridae, but it clustered with 12 species of Styelidae into a clade. Based on this, it can be suggested that YZ‐2024 is a new species of sea squirt in the northern Beibu Gulf of the South China Sea. Moreover, this study is the first report of molecular identification of sea squirt species in the northern Beibu Gulf of the South China Sea.

## Abstract 2

The morphological and molecular characteristics of the new sea squirt distinguish it from other sea squirts. Its mitochondrial genome size was 14,520 bp, and the proportions of bases A, T, G, and C comprising 26.83%, 47.16%, 16.91%, and 9.10%, respectively. It clusters with its congeneric species *Microcosmus sulcatus*. This study is the first report of molecular identification of sea squirt species in the Beibu Gulf of the South China Sea.

**Abbreviations**
atp6: ATP synthase F0 subunit 6
atp8: ATP synthase F0 subunit 8
BLAST: Basic Local Alignment Search Tool
cox1: cytochrome *c* oxidase subunit 1
cox2: cytochrome *c* oxidase subunit 2
cox3: cytochrome *c* oxidase subunit 3
cytb: cytochrome *b*
mtDNA: mitochondrial DNA
nad1: NADH dehydrogenase subunit 1
nad2: NADH dehydrogenase subunit 2
nad3: NADH dehydrogenase subunit 3
nad4: NADH dehydrogenase subunit 4
nad4l: NADH–ubiquinone/plastoquinone oxidoreductase, chain 4 L
nad5: NADH dehydrogenase subunit 5
nad6: NADH dehydrogenase subunit 6
NCBI: National Center for Biotechnology Information
PCGs: protein‐coding genes
RSCU: relative synonymous codon usage
TDRL: tandem duplication‐random losses

## 1 Introduction

Sea squirts, belonging to the phylum Chordata, subphylum Urochordata, and class Ascidiacea, are the closest relatives of vertebrates (Bourlat et al. 2006; Delsuc et al. 2006; Swalla and Smith 2008). Over 2000 years ago, tunicates were recorded as invertebrates until 1866 when the Russian scholar Kowalevsky, after careful study of their embryonic development and metamorphosis, officially classified them under the phylum Chordata (Liu and Zheng 2009). Ascidians occupy the evolutionary position at the boundary of invertebrates and vertebrates, exhibit adaptation to broad environmental conditions and are distributed globally (Goldstien et al. 2011; Wei et al. 2020). Sea squirts constitute the major group in the subphylum Urochordata, accounting for over 90% of all species. Sea squirts are a major community in the benthic marine ecosystem and contribute significantly to biodiversity through their distribution on several natural and artificial substrata across the world (Shenkar and Swalla 2011). Due to their unique evolutionary morphology, sea squirts play a significant role in the study of animal evolution, development, and the origin of chordates (Dahlberg et al. 2009).

Common species of sea squirts include *Styela clava*, *Ciona intestinalis*, and *Botrylloides violaceus*, with *C. intestinalis* having a widespread distribution worldwide (Liu and Zheng 2009; Goldstien et al. 2011; Wei et al. 2020). Sea squirts exist either as solitary individuals or in colonies, living attached to substrates underwater. Individual species can reach lengths of up to 200 mm, while colony lengths can exceed 0.5 m. Adult ascidians are often found attached to natural substrates such as coral reefs, rocky substrates, and living shellfish shells (Coffey 2001; Lambert 2001, 2005a, 2005b; Liu and Zheng 2009), and artificial substrates such as marina floats, boat hulls, pilings, and aquaculture facilities (e.g., kelp rafts, scallop cages; Lambert and Lambert 2003; Castilla et al. 2002; Lambert 2002, 2004; Turon et al. 2003). Ascidians have a major impact on the regional marine biodiversity and on aquaculture industries (Wei et al. 2020). Ascidians are a bioindicator of metals in a marine ecosystem (Carman, Bullard, and Donnelly 2007; Radhalakshimi, Sivakumar, and Ali 2014), and a suitable model organism to evaluate the toxicity of marine contaminants and for seawater quality assessments and reprotoxicological bioassays (Gallo et al. 2011, 2016; Gallo and Tosti 2015a, 2015b; Gallo 2017; Beyer et al. 2023).

In China, 66 species of sea squirts have been recorded, including 5 species in the Bohai Sea, 21 species in the Yellow Sea, 24 species in the East China Sea, and 53 species in the South China Sea (Zheng 1995). In the South China Sea, the most recorded species of sea squirts are found, with the family Pyuridae alone comprising species such as *Herdmania momus* , *Microcosmus australis*, *M. erasperatus*, *Pyura lignosa* , *Pyura sp*., and *P. vittata* . Among them, *M. australis* and *M. erasperatus* are dominant species in the waters surrounding Hainan Island, the Pearl River Delta, and the western Guangdong Province (Zheng 1995).

As is well known, the classification and identification of sea squirts are extremely challenging, mostly relying on morphological features, leading to misidentifications of ascidian species frequently (Monniot, Monniot, and Laboute 1991; Mastrototaro and Dappiano 2008; Lambert 2009). Initially, ascidians were classified as mollusks by Linnaeus and the French zoologist Georges Cuvier (1769–1832; Christiaen et al. 2009), but it was not until 1866 that Kovalevsky recognized the chordate characteristics of larval tunicates (Irvine, Ristoratore, and Di Gregorio 2019). Savigny also recognized the ascidians as a distinct group separate from the Mollusca (Savigny 1816; Lambert 2005a, 2005b). In cases where traditional morphology‐based species identification and classification methods fail, molecular identification serves as an effective means, shedding light on taxonomy (Geller, Darling, and Carlton 2010; Rubinstein et al. 2013). Indian researchers have conducted molecular identifications of various local sea squirt species. For instance, Lyappan used COI gene sequences to identify sea squirts ( *Didemnum candidum* , *Ascidia ahodori*, and *Styela clava* ) in the Parlk Bay region of southeastern India (Lyappan, Ananthan, and Sathishkumar 2016). Sri Kumaran identified two sea squirt species, *Didemnum granulatum* (JQ013198) and *Didemnum psammatodes* (JN624758), along the Tuticorin coast (Sri Kumaran, Bragadeeswaran, and Meenakshi 2014). Jaffar Ali and Ahmed reported for the first time the COI gene sequences of two solitary sea squirt species, *Herdmania momus* Savigny 1816, and *Microcosmus squamiger* Michaelsen, 1927, from the Thoothukudi Coast in India (Jaffar Ali and Ahmed 2016). Scientists from Israel, France, Italy, and the United States utilized Sanger sequencing to obtain mitochondrial genomes of *Rhodosoma turcicum* (Corellidae), *Ascidiella aspersa* (Ascidiidae), *Botrylloides aff. leachii* (Styelidae), *Polycarpa mytiligera* (Styelidae), *Halocynthia spinosa* (Pyuridae), and *Pyura gangelion* (Pyuridae), providing methodological references for the classification and identification of other sea squirt species (Rubinstein et al. 2013).

In September 2023, 50 sea squirt specimens were collected from scallop farming cages along the coast of Xilian Town, Xuwen County, Zhanjiang City, in the northern Beibu Gulf of the South China Sea. The external morphological characteristics and internal structure of the specimens were observed, and their DNA was extracted and sequenced to obtain mitochondrial genome information; based on this, its evolutionary status is analyzed. The results of this study will enrich genetic information of ascidians, and they are immediately valuable for developmental biology of ascidians.

## 2 Materials and Methods

### 2.1 Sample Collection

**FIGURE 1:**
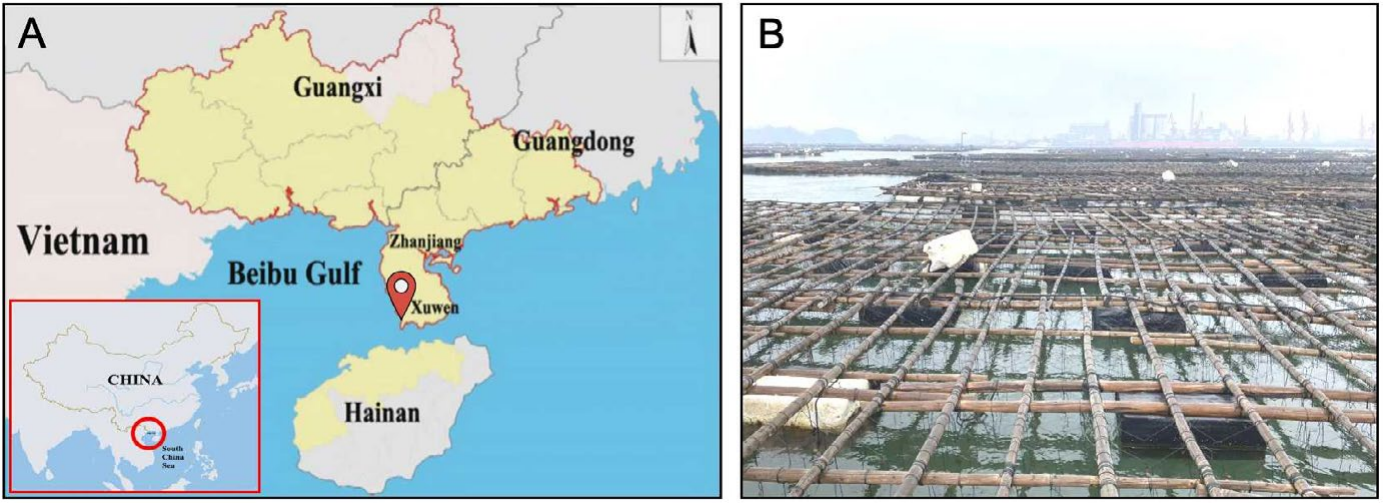
Sample collection information of *Microcosmus* sp. z YZ‐2024. (A) The red circle indicates the sampling area; the red pen indicates the sampling point. (B) Habitat of *Microcosmus* sp. z YZ‐2024.

**FIGURE 2:**
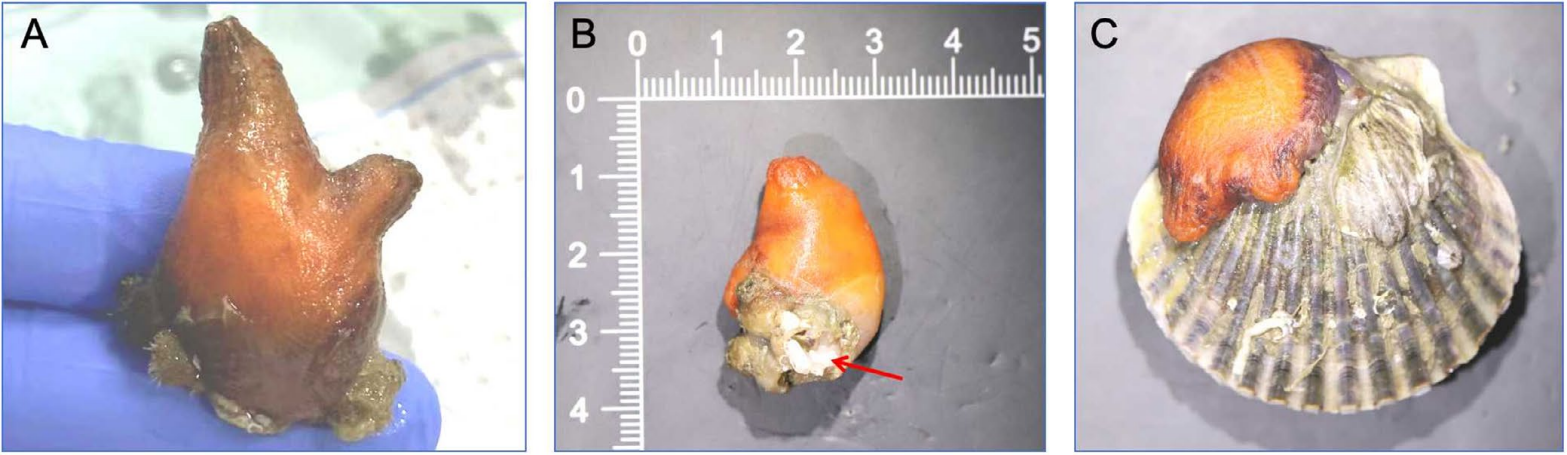
Reference image of *Microcosmus* sp. z YZ‐2024. The photo was taken by Yichuan Zhang on September 20, 2023 in Xuwen County, Zhanjiang City, Guangdong Province, China. (A) Lateral view. (B) Ventral view: the red arrow points to the mantle muscle. (C) The morphology of *Microcosmus* sp. z YZ‐2024 when attached.

Samples were collected from scallop farming cages along the coast of Xilian Town, Xuwen County, Zhanjiang City, Guangdong Province, in the northern Beibu Gulf of the South China Sea, (20.402422°N, 109.888322°E; Figure 1A). Sea squirts, along with their attachment substrates (Figures 1B and 2C), were removed and placed in a fiberglass tank of 100 L, filled with still water, and brought back to the laboratory. After 1 day of acclimation in the laboratory recirculating aquaculture system, morphological observations were conducted on the samples.

### 2.2 Internal Structure Observation

The sea squirt was fixed in a 4% paraformaldehyde solution for over 24 h. The sample was then dehydrated using different grades of ethanol (70%, 80%, 90%, and 100%). After clearing the sample with xylene, it was placed in a 60°C paraffin bath for at least 1 h. The sample was then embedded in a cooled solid paraffin mold and sectioned into 4‐μm‐thick slices using a paraffin microtome (LEICA RM2016). After staining with hematoxylin–eosin, the slices were observed under a microscope (Nikon, ECLIPSE CI‐L, Japan) to examine the sample's internal structural characteristics at a magnification of 10 × 4 and 10 × 10, and images were captured using a Nikon digital camera DS‐FI3 system.

### 2.3 DNA Extraction and Gene Fragment Amplification

Muscle tissue from the pharynx of sea squirts was washed and immediately stored at −80°C. Genomic DNA was extracted following the manufacturer's instructions (Jiangsu CoWin Biotech Co. Ltd.), and DNA quality was assessed using 0.8% agarose gel electrophoresis and a Nanodrop spectrophotometer (Thermo Fisher Scientific, USA). Partial sequences of the mtDNA COI gene were amplified using specific primers: LCO1490F: 5′‐GGT CAA CAA ATC ATA AAG ATA TTG G‐3′ and HCO2198R: 5′‐TAA ACT TCA GGG TGA CCA AAA AAT CA‐3′. PCR was performed in a 25‐μL reaction mixture containing 12.5 μL PCR Mix (TaKaRa PCR Amplification Kit, Takara, Dalian, China), 0.5 μL each forward and reverse primer (10 μM), 2 μL DNA template (50 ng/μL), and an aliquot of sterile water to attain the total volume. The PCR amplification conditions were as follows: 94°C for 5 min for 1 cycle; 35 cycles of 94°C for 1 min, 51°C for 1 min, 72°C for 1 min, followed by a final extension at 72°C for 10 min.

The PCR products were analyzed by 1.5% agarose gel electrophoresis, and the target bands were identified under ultraviolet light, excised, and recovered using the Gel DNA Recovery Kit (DP209) (Tiangen Biotech (Beijing) Co. Ltd.) according to the manufacturer's instructions. The recovered products were dissolved in 20 μL of sterile deionized water and sent to Guangzhou Science Corporation of Gene for sequencing.

### 2.4 Library Construction and High‐Throughput Sequencing

After passing quality control, the samples were subjected to the Illumina DNA library construction standard procedure to generate paired‐end sequencing libraries with insert sizes of 350 bp. Following library construction, quality control was performed using qPCR and the Agilent 2100 Bioanalyzer (Agilent Technologies, USA). DNA libraries that passed quality control were sequenced using the high‐throughput sequencing platform Illumina Novaseq6000.

### 2.5 Mitochondrial Genome Sequence Assembly

The mitochondrial genome sequences were assembled using SPAdes v.3.15.2 (Bankevich et al. 2012; Lapidus et al. 2014). Initial annotation of the mitochondrial genome was conducted using MITOS2 (http://mitos2.bioinf.uni‐leipzig.de/index.py) and ORF Finder (https://www.ncbi.nlm.nih.gov/orffinder/). The preliminary annotation results were validated and corrected by comparing them with the mitochondrial genome sequences of closely related species using Blastp and Blastn methods (https://blast.ncbi.nlm.nih.gov/Blast.cgi). The circular structure of the mitochondrial genome was visualized using Proksee (https://proksee.ca/). PhyloSuite v1.2.0 was utilized for the analysis and calculation of nucleotide composition, compositional skewness, codon usage, and relative synonymous codon usage (RSCU) of protein‐coding genes (PCGs). AT‐skew = (A − T)/(A + T), GC‐skew = (G − C)/(G + C).

### 2.6 Construction of Mitochondrial Phylogenetic Tree

Species within the same order (Stolidobranchia) with complete coding genes were downloaded from NCBI for the construction of the phylogenetic tree. Phylogenetic analysis of Stolidobranchia was carried out using PCG nucleotide sequences from 23 species. *Clavelina lepadiformis* (NC_012887.1) mitochondrial genome, belonging to the same class but in a different order, was chosen as the outgroup. PCGs from the mitochondrial genomes of selected species used for phylogenetic tree construction were extracted individually (selecting genes common to all species for tree construction). Based on MUSCLE v.3.8.31, each of the PCG nucleotide sequences from all species was individually aligned (http://www.drive5.com/muscle/; Edgar 2004) and then aggregated into a sequence matrix to construct the phylogeny. The maximum likelihood method (ML) was employed for constructing the phylogenetic tree using IQ‐TREE 2.0.5 (http://www.iqtree.org/; Minh et al. 2020), with tree construction parameters set as ‐m MFP ‐B 1000 ‐alrt 1000.

### 2.7 Gene Arrangement Analysis

To further explore the differences in gene arrangement between YZ‐2024 and other sea squirts, GenBank files of the aforementioned species used for phylogenetic tree construction were downloaded from NCBI to obtain the gene arrangement information for each species. The iTOL (https://itol.embl.de/upload.cgi) website was utilized to generate graphical representations of the gene arrangements for each species. This facilitated the intuitive display of gene arrangement variations among different species.

## 3 Results

### 3.1 External Morphology

The adult individual resembles a tunica, the body length rarely exceeds 6 cm, tapering gradually toward the tail. Except for the attachment site, the tunica is orange‐red (Figure 2). YZ‐2024 often attaches to other marine organisms such as scallops and oysters (Figure 2C), with two modes of attachment: one involves attaching with the base using the mantle muscles, while the other involves attaching by pressing the entire body against the substrate with its lateral surface. The YZ‐2024 individual features a bifurcated tail, resulting in two distinct tails of significantly different sizes (Figure 2A). The buccal opening is located at the tip of the larger tail, while the exhalent aperture is positioned at the end of the smaller tail. Typically, the exhalent aperture and buccal opening perform their respective functions independently, yet under external stimuli, both of them simultaneously expel water. This is an intriguing phenomenon discovered in this study. The surface of the tunica is smooth and soft, without protuberances. When attached to the substrate, it adheres to other objects through the pallial muscle, which consists of 3–4 white loose muscle bundles (Figure 2B). Often, epibionts can be found on the tunica near the attachment site.

### 3.2 Internal Structure

**FIGURE 3:**
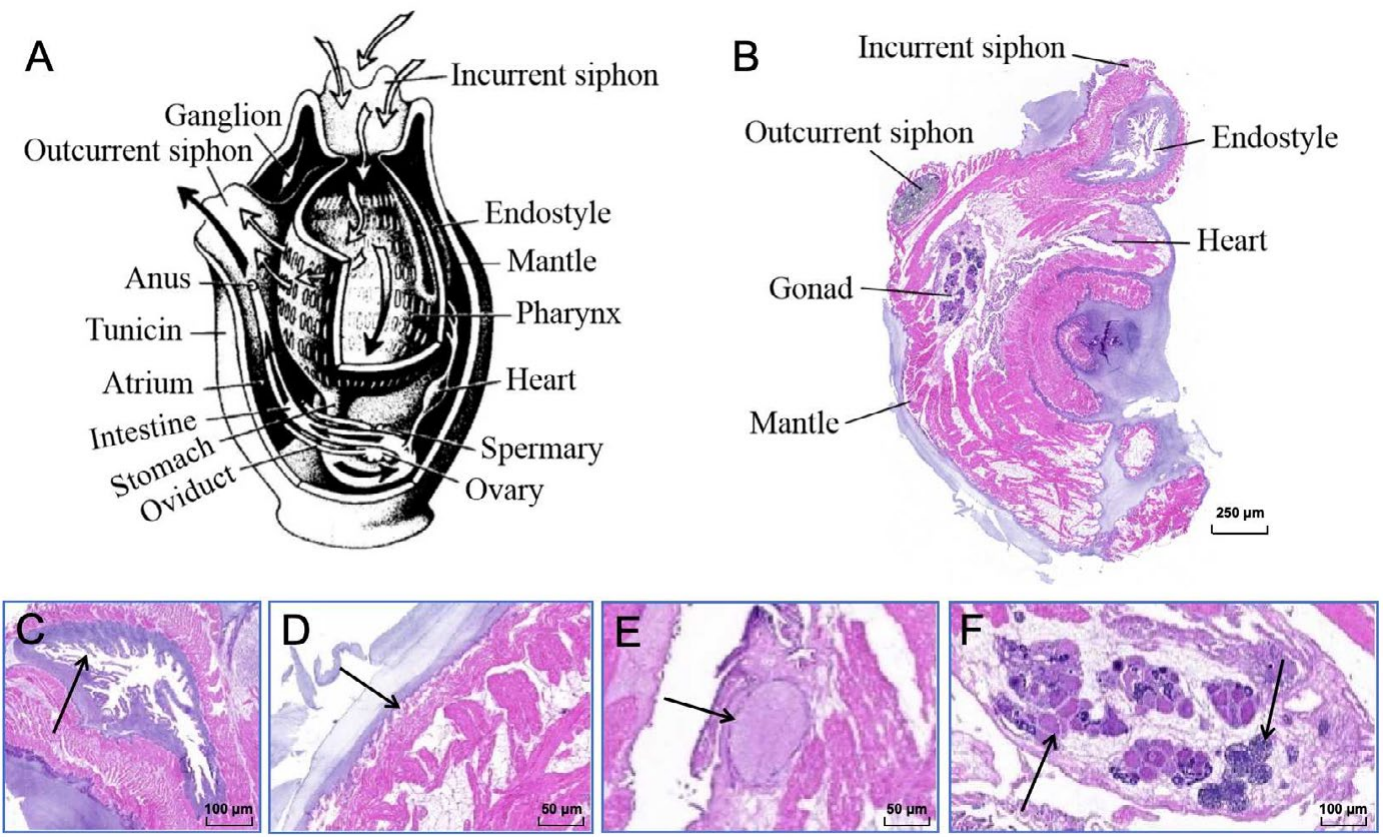
The internal structure of the sea squirt. (A) Schematic diagram of inner organs of sea squirt (Charles and Gretchen Lambert 2003a, 2003b). (B) Internal structure of *Microcosmus* sp. z YZ‐2024. (C) Endostyle of *Microcosmus* sp. z YZ‐2024, the arrow points to the cilia; (D) mantle; (E) heart; (F) gonad; blue arrow indicates the testicles; black arrow points to the egg cell.

Figure 2 shows the internal structure of the sea squirt. With reference to the structural model diagram of the sea squirt (Figure 3A), incurrent siphon, outcurrent siphon, endostyle, mantle, heart, gonad, sperms and egg cells of YZ‐2024 can be clearly found (Figure 3B). The nervous system and sensory organs of YZ‐2024 are highly degenerated, with no ganglia observed. The tunica does not contract abruptly when disturbed. The endostyle is located below the buccal opening and contains ciliated cells. The column wall displays irregularly arranged cilia, whose continuous movement directs water flow (Figure 3C). The endostyle's terminal end connects to the esophagus. As an invertebrate, the sea squirt relies on seawater to maintain the balance of internal and external osmotic pressure, with the endostyle also serving the function of filling with seawater to maintain this balance and support the body. The heart is located in the pericardial cavity on the ventral side of the body and is oval‐shaped (Figure 3E). The intestine is curved with folds on the intestinal wall, and food particles can be clearly seen inside the intestine as they enter from the stomach and are absorbed by the intestine. Unabsorbed food is also expelled through this route.

YZ‐2024 is hermaphroditic, with testicles of varying shapes closely packed together, surrounding the eggs, and a few testicles and eggs arranged in an interspersed manner. The ovary is irregularly shaped and contains many spherical oocytes, with primordial oocytes arranged closely (Figure 3F). The outer surface of the mantle is covered by an ectoderm layer of epithelial cells, interspersed with muscle fibers derived from the mesoderm, which control the expansion, contraction, opening, and closing of the body as well as the buccal opening and exhalent aperture (Figure 3D).

### 3.3 Structure of the Complete Mitochondrial Genome

**FIGURE 4:**
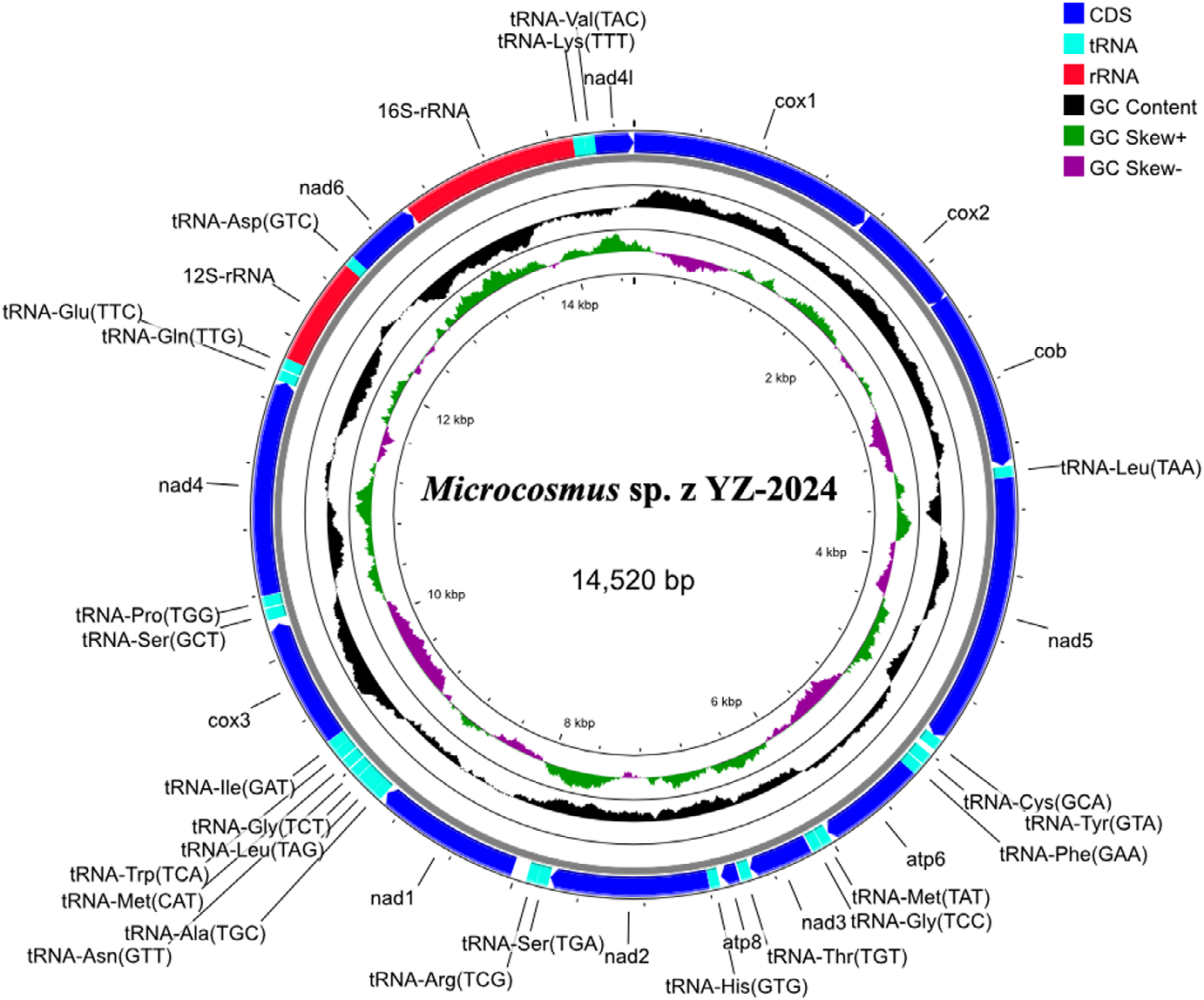
The complete mitochondrial genome of *Microcosmus* sp. z YZ‐2024. The circular structure of the mitochondrial genome of *Microcosmus* sp. z YZ‐2024. The inner circle represents GC skew. The middle circle represents GC content. The outer circle shows the gene distribution—blue for CDS, red for rRNA, and aqua for tRNA.

The complete mitochondrial genome of YZ‐2024 is 14,520 bp in length (GenBank accession number: PP067884), shorter than that of its congeneric species *Microcosmus sulcatus* (15,028 bp, NC 013752), and the species in the same family, that is, *Pyura mirabilis* (15,778 bp, NC 079570) and *Herdmania momus* (15,816 bp, NC 013561), but longer than *Pyura gangelion* (14,418 bp, NC 021465), which is also in the same family. The base composition is A (26.83%), T (47.16%), C (9.10%), and G (16.91%), with a G + C content ratio of 26.0% and an A + T content ratio of 74.0%. The GC ratio is lower than that of its congeneric species *M. sulcatus* (29.78% of G + C and 70.22% of A + T). The mitochondrial genome of YZ‐2024 contains 13 PCGs, 22 tRNA genes, and 2 rRNA genes (one 12S rRNA and one 16S rRNA). The circular structure of the mitochondrial genome of YZ‐2024 is shown in Figure 4.

### 3.4 Protein‐Coding Genes

There are 13 PCGs in the mitochondrial genome of YZ‐2024, with a total length of 10,884 bp, accounting for 74.96% of the complete mitogenome. Their sizes range from 105 bp (atp8) to 1692 bp (nad5). In YZ‐2024, these PCGs utilize two different triplets as the start codons, including ATG and GTG. Among them, 11 PCGs (cox1, cox2, cox3, nad1, nad2, nad3, nad4, nad5, atp6, atp8, and cytb) use ATG as start codon, nad6 and nad4l use GTG as start codon. These 13 PCGs contain two different types of stop codons: six PCGs (cox1, cox2, cox3, nad3, atp8, and nad4) employ TAA as stop codon, while seven PCGs (nad1, nad2, nad4l, nad5, nad6, atp6, and cytb) employ TAG as stop codon (Table 1). Notably, the usage of both TAA and TAG as stop codons is common in animals.

**TABLE 1:** Information of the coding genes in *Microcosmus* sp. z YZ‐2024 mitochondrial genome.

Gene/element	Strand	From	To	Size(bp)	GC percent	Inferred initiation codon	Inferred termination codon	Anticodon	Intergenic nucleotide (bp)
cox1	+	1	1542	1542	31.00%	ATG	TAA		3
cox2	+	1546	2232	687	30.42%	ATG	TAA		−11
cob	+	2222	3325	1104	29.44%	ATG	TAG		7
tRNA‐Leu	+	3333	3400	68	35.29%			TAA	3
nad5	+	3404	5095	1692	24.88%	ATG	TAG		−1
tRNA‐Tyr	+	5095	5164	70	34.29%			GTA	38
tRNA‐Cys	+	5203	5275	73	36.99%			GCA	8
tRNA‐Phe	+	5284	5353	70	34.29%			GAA	0
atp6	+	5354	5995	642	26.17%	ATG	TAG		5
tRNA‐Met	+	6001	6064	64	14.06%			TAT	4
tRNA‐Gly	+	6069	6132	64	20.31%			TCC	10
nad3	+	6143	6529	387	24.29%	ATG	TAA		3
tRNA‐Thr	+	6533	6601	69	18.84%			TGT	10
atp8	+	6612	6716	105	23.81%	ATG	TAA		17
tRNA‐His	+	6734	6796	63	22.22%			GTG	3
nad2	+	6800	7786	987	21.68%	ATG	TAG		3
tRNA‐Ser	+	7790	7861	72	23.61%			TGA	0
tRNA‐Arg	+	7862	7926	65	27.69%			TCG	97
nad1	+	8024	8938	915	25.36%	ATG	TAG		8
tRNA‐Ala	+	8947	9009	63	17.46%			TGC	−1
tRNA‐Leu	+	9009	9073	65	26.15%			TAG	−3
tRNA‐Gly	+	9071	9136	66	19.70%			TCT	4
tRNA‐Asn	+	9141	9203	63	23.81%			GTT	9
tRNA‐Met	+	9213	9276	64	23.44%			CAT	8
tRNA‐Trp	+	9285	9349	65	24.62%			TCA	1
tRNA‐Ile	+	9351	9416	66	31.82%			GAT	3
cox3	+	9420	10,205	786	32.06%	ATG	TAA		34
tRNA‐Ser	+	10,240	10,311	72	31.94%			GCT	9
tRNA‐Pro	+	10,321	10,389	69	27.54%			TGG	4
nad4	+	10,394	11,728	1335	23.82%	ATG	TAA		−4
tRNA‐Glu	+	11,725	11,790	66	22.73%			TTC	9
tRNA‐Gln	+	11,800	11,866	67	20.90%			TTG	0
12S‐rRNA	+	11,867	12,536	670	24.33%				0
tRNA‐Asp	+	12,537	12,603	67	31.34%			GTC	0
nad6	+	12,604	13,047	444	19.37%	GTG	TAG		0
16S‐rRNA	+	13,048	14,144	1097	22.61%				0
tRNA‐Lys	+	14,145	14,217	73	30.14%			TTT	0
tRNA‐Val	+	14,218	14,279	62	17.74%			TAC	−17
nad4l	+	14,263	14,520	258	22.87%	GTG	TAG		0

**FIGURE 5:**
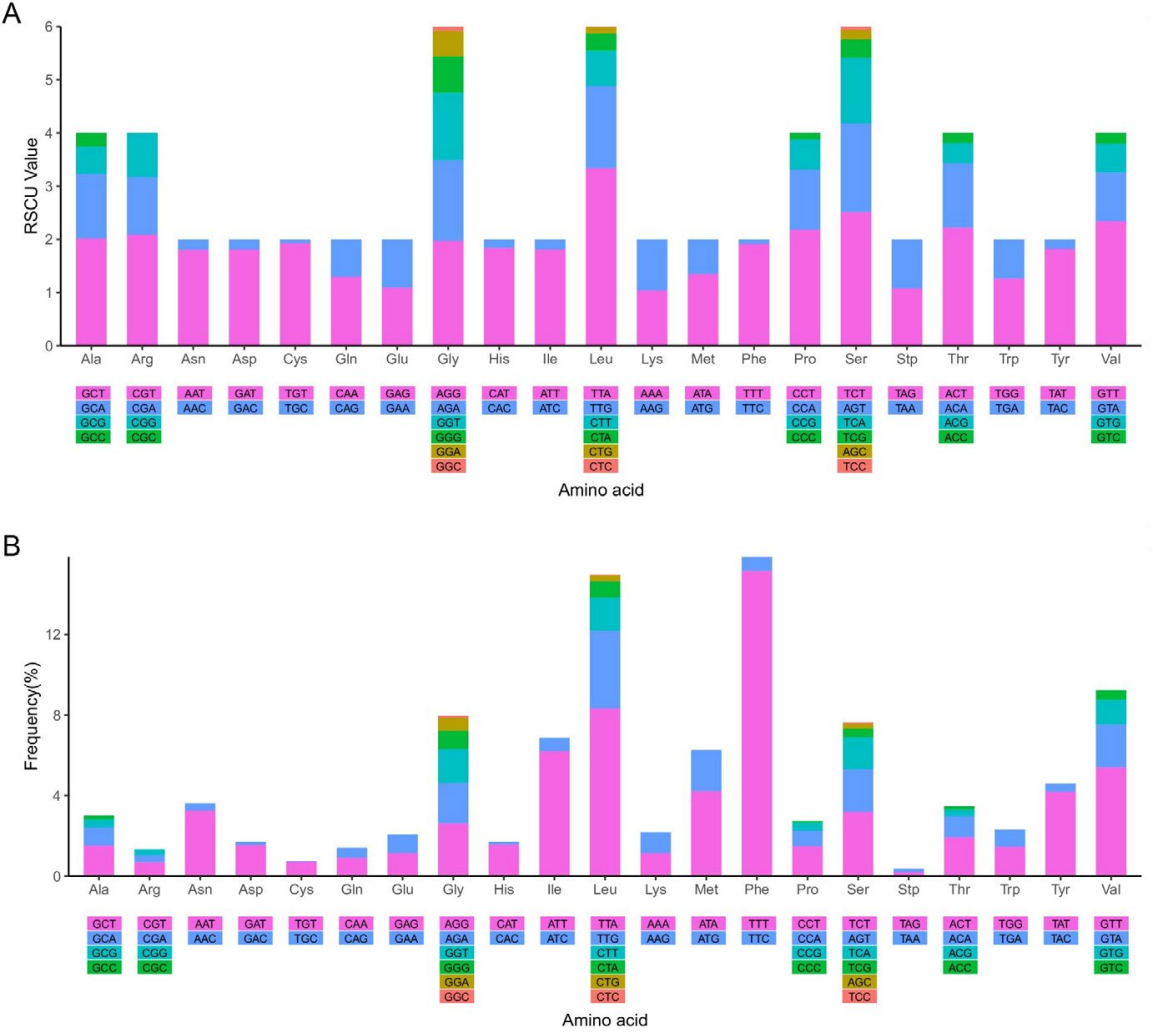
Relative synonymous codon usage (RSCU) (A) and amino acid use frequency (B) in *Microcosmus* sp. z YZ‐2024 mitogenome.

The GC skew of all PCGs is positive, while the AT skew is negative, indicating a preference for thymine. The relative synonymous codon usage (RSCU) distribution in the mitochondrial genome of YZ‐2024 reveals that the RSCU values of glycine (Gly), leucine (Leu), and serine (Ser) are highest, and the codons encoding Gly, Leu, and Ser were most abundant—they are encoded by six codons. Ala, Arg, Pro, Thr, and Val are encoded by four codons each, and the remaining amino acids are encoded by two codons. Whereas, codons for Gly (GGC) and Ser (TCC) were rare. Among the codons utilized, TTT‐Phe, TTA‐Leu, TCT‐Ser, and GTT‐Val are the most frequently employed. Particularly, TTT and TTA emerged as the most preferred codon (Figure 5).

### 3.5 Mitochondrial Phylogenetic Tree

**FIGURE 6:**
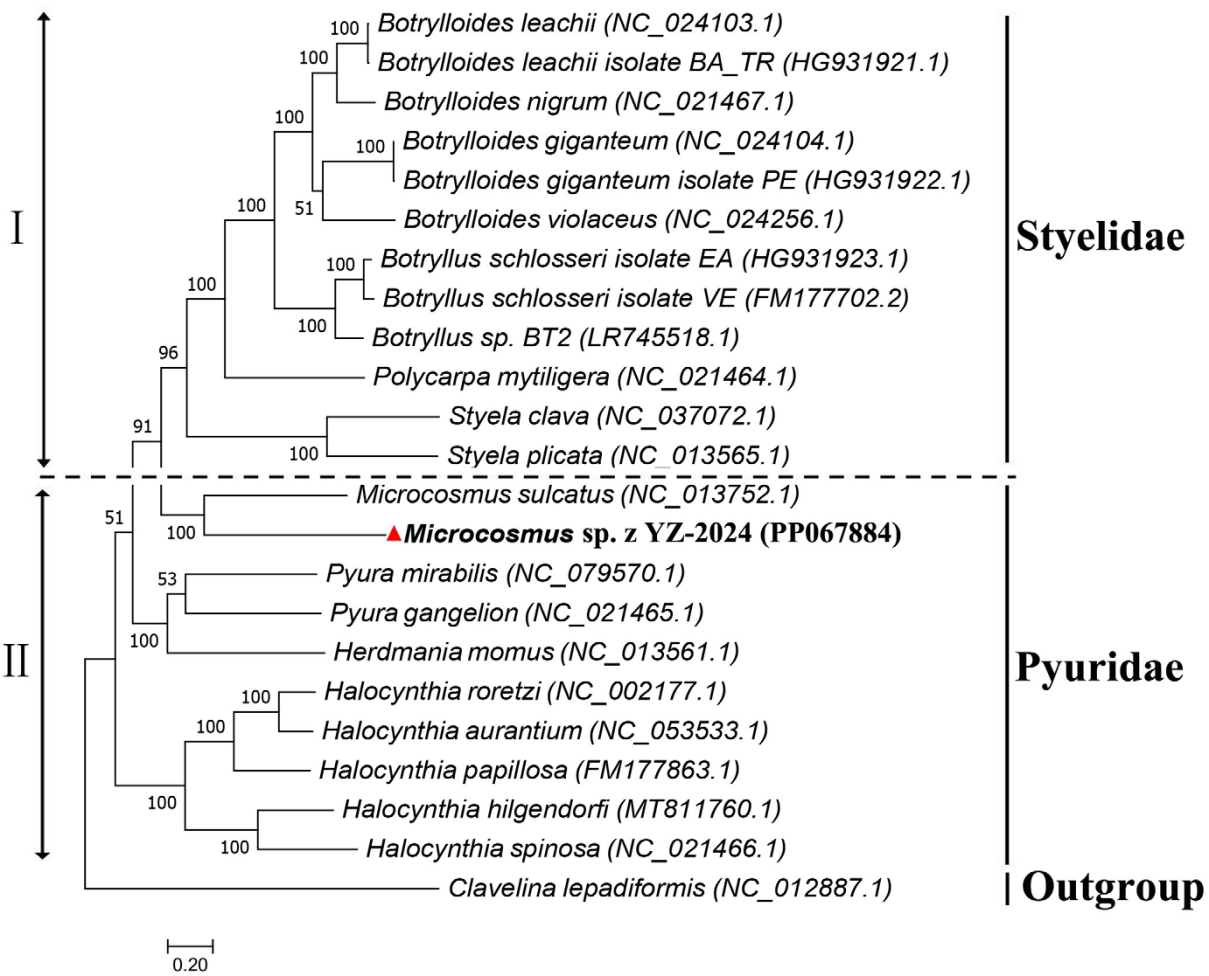
Phylogenetic relationship analysis based on the mitochondrial genomes of 23 sea squirt species. *Clavelina lepadiformis* is presented in the form of outgroups.

The genes used for constructing the phylogenetic tree in this study were the 12 conserved mitochondrial PCGs shared by all species included in the tree, namely COX2, COX3, CYTB, COX1, ATP6, ND4L, ND1, ND3, ND2, ND5, ND4, and ND6. The constructed phylogenetic tree is shown in Figure 6. The results indicate that YZ‐2024 clustered first with its congeneric species *M. sulcatus* , suggesting a closer phylogenetic relationship between the two, and the genetic distance between them is 0.726. Furthermore, it is closer to *S. clava* and *Styela plicata* of the genus Styela in the family Styelidae, followed by *Pyura mirabilis* and *Pyura gangelion* of the genus *Pyura* in the family Pyuridae, indicating a close phylogenetic relationship between YZ‐2024 and the aforementioned five sea squirt species. Additionally, the phylogenetic tree is divided into two branches (I and II; Figure 6). Branch I belongs to Styelidae, showing monophyletic evolution and including four genera (Botrylloides, Botryllus, Polycarpa, and Styela), and having high bootstrap value. Branch II belongs to Pyuridae, showing polyphyletic evolution and containing four genera (Microcosmus, Pyura, Herdmania and Halocynthia).

### 3.6 Mitochondrial Gene Arrangement

**FIGURE 7:**
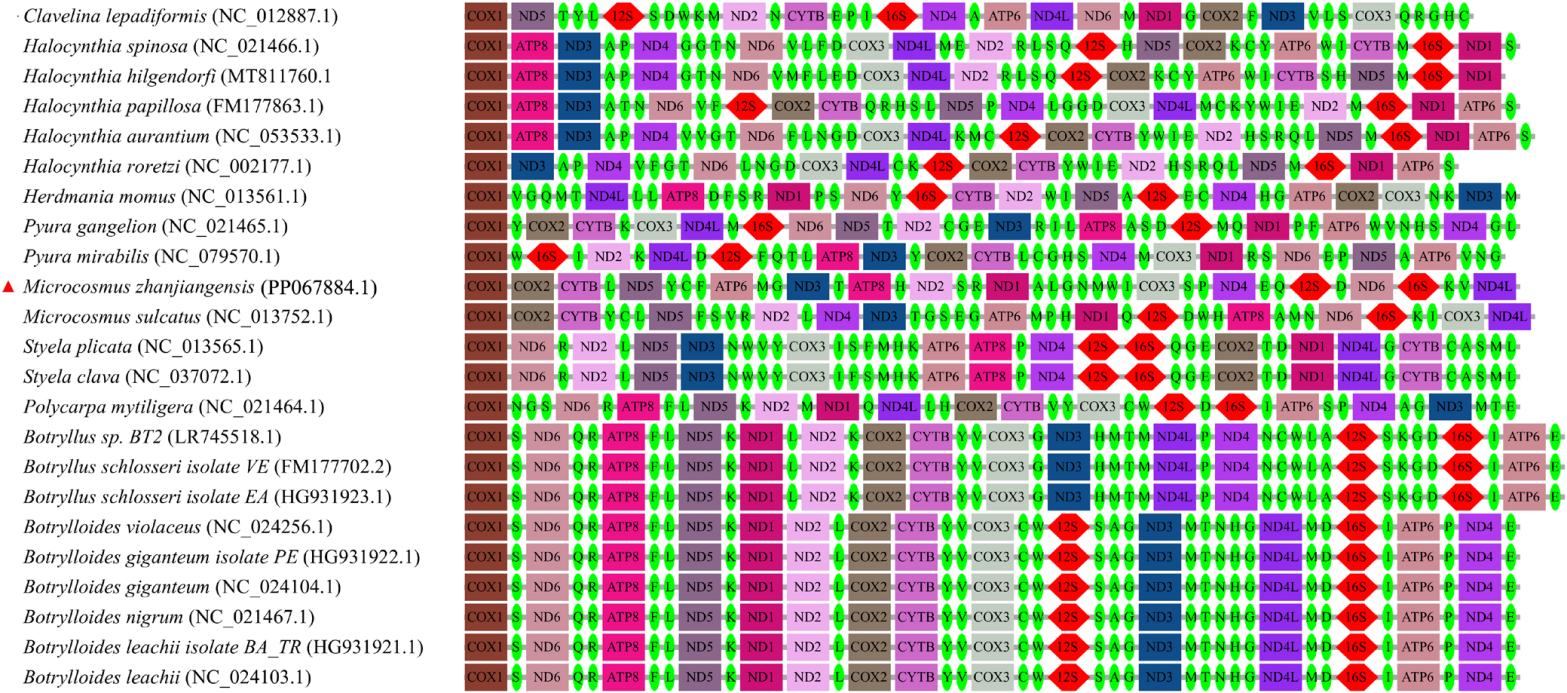
Mitochondrial gene arrangements of 23 sea squirt species.

The characteristics of gene arrangement include translocation, inversion, and reverse translocation. The gene order of the 23 mitochondrial genomes is illustrated in Figure 7, with conserved gene blocks marked in different colors. Except for COX1, the gene order of the mitochondrial genome of YZ‐2024 differs from that of the other 22 species. The genes COX2, CYTB, and ND4L of YZ‐2024 are in the same positions as those of its congeneric species *M. sulcatus* . Compared to *M. sulcatus* , tRNA Leu and ND5 undergo translocation upstream. Additionally, tRNA Pro and NAD4 are adjacent and have a 5‐bp overlap. Furthermore, the distribution of the tRNA Pro‐NAD4 gene block covers distantly related species on the phylogenetic tree (Figure 6). The mitochondrial gene arrangement information above indicates that YZ‐2024 is a new species of sea squirt. In addition, the result of mitochondrial gene arrangement shows that species of Styelidae (except *S. clava* , *S. plicata*, and *Polycarpa mytiligera*) have relatively regular gene order, while the gene order of species in Pyuridae is disorganized and irregular. In this study, the gene tRNA Leu and ND5 of YZ‐2024 are a whole, and their position is basically consistent with that of *S. clava* and *S. plicata* , which belong to Styelidae. Meanwhile, COX2 and CYTB are tied together, which is consistent with most species of Styelidae. This result is consistent with that of phylogenetic tree analysis.

## 4 Discussion

Traditional taxonomy typically classifies animals and plants based on their morphological features, which are important indicators of species diversity and are widely used in scientific research fields such as biological taxonomy, phylogenetics, and evolutionary biology. In the early stages of taxonomic research, morphological methods played a significant role in species identification and classification (Fei, Hu, and Ye 2009). Alexander Koval evsky (1840–1901) classified ascidians as bona fide chordates based on their characteristics of morphology and inner structure (Delsuc et al. 2006). The *Chinese Economic Animal Fauna* published in 1963 provided a detailed description of the morphological characteristics of *C. intestinalis* produced in Qingdao (Zhang, Zhang, and Wu 1963). Zheng (1984) reviewed the species and distribution of sea squirts along the Chinese coast, describing the morphological characteristics of dominant species such as *Styela clava, Ciona intestinalis*, *Molgula manhattensis* , and *Styela plicata* . Ge and Zang reported on the *Botrylloides* and *Styela clava* in the Jiaozhou Bay area in 1983 and 1987, including three new species (*Styela* sp., *Cnemidocarpa* sp. and Botryllus tsingtaoensis n.sp*.) (Ge and Zang 1983, 1987). Li, Yin, and Chen (2015) reported on the morphological characteristics of a newly recorded species of Pyrosomatidae in the South China Sea. Zhang (1986) identified fossil Appendicularians based on morphological features.

In this study, YZ‐2024 exhibits unique external morphological characteristics: (1) The body length of YZ‐2024 is less than 6 cm, which is different from other sea squirts, such as *Styela clava* (average: 83 mm; Ge and Zang 1987), *Botrylloides violaceus* Saito 1981 (3–4 mm; Ge and Zang 1983), *Ciona intestinalis* (≤ 5 mm; Liu 2005), *Styela sinensis* (≤ 10 mm), *Botryllus schlosseri* (1 mm; Zhang, Fang, and Dong 2000). (2) The tunica surface of YZ‐2024 is smooth without tubercles. *Styela sp*., *Styela sinensis n. sp*., and *Styela canopus* Savigny 1816 collected in Jiaozhou Bay (Yellow Sea, China) also have the same characteristics (Ge and Zang 1987), whereas most sea squirts have tubercular projections on their bodies and the tunica is rough and firm, such as *Cnemidocarpa sp*., *Cnemidocarpa chinensis* Tokioka 1967 (Ge and Zang 1987; Zhang, Che, and Xu 2005; Lyappan, Ananthan, and Sathishkumar 2016), and *Ciona intestinalis* (Li et al. 2024), which is a distinctive distinguishing feature of this species; (2) apart from the attachment site, the tunica is orange‐red in color, differing from reported species in the same family, such as *Microcosmus erasperatus* (purple‐red or brown‐red), *Pyura vittata* (Stimpson, 1852; red), *Herdmania momus* (Savigny 1816; milky white or pale red tunica; Zheng 1984), and *Cnemidocarpa amphora* (Kott, 1992; light or dark brown; AlQurashi and Ibrahim 2023).

The internal structure of animals is also an important basis for taxonomy, which can provide direct evidence for species identification (Behura 2006; Frézal and Raphaël 2008; Patwardhan, Ray, and Roy 2014). YZ‐2024 exhibits internal structural feature: (1) No neural ganglia were found, and the nervous system and sensory organs are highly degraded. Upon disturbance, the tunica does not abruptly contract. Previous research reported that ascidian tunicate larvae have an extremely simple nervous system, with only 177 neurons in the brain of *C. intestinalis* (Ryan, Lu, and Meinertzhagen 2016). The nervous system of *C. intestinalis* is degraded, and the nerve center is only a nervus ganglion (Liu 2005; Zhou 2008). (2)The heart is oval‐shaped. The ascidian heart is a model for the morphogenesis of a simple chordate organ (Irvine, Ristoratore, and Di Gregorio 2019). The heart of *C. intestinalis* is peach‐shaped (Zhou 2008). These external morphological characteristics and internal structural feature indicate that the sampled specimens represent a new species of sea squirt.

Morphological characteristics of animals can be influenced by environmental factors, genetic mutations, and other factors, leading to inaccurate classification results (Stefaniak et al. 2012). Moreover, the phenomenon of convergent evolution among species leads to the complexity of morphological evolution, making it difficult to ensure the accuracy of species identification results (Nevo 2001). With the rapid development of molecular biology, species identification and classification have become simpler, more accurate, and more reliable (Yang and Rannala 2010). Mitochondrial DNA, as relatively independent genetic material outside the cell nucleus, is characterized by easy isolation and purification, small molecular weight, simple structure, and fast evolution rate. It is widely used in species identification and classification research (Saccone et al. 2000). Li et al. (2023) combined morphological characteristics and molecular features to identify a new species of loach in Guangxi. Ma et al. (2010) identified a new species of *Ciona intestinalis* , *C. savignyi* , in Qingdao and Dalian, China, by combining morphology and mitochondrial cytochrome *b* (Cyt *b*) gene sequences. Lyappan, Ananthan, and Sathishkumar (2016) used COI sequences to identify four species of sea squirts in the Parlik Bay area of southeastern India, including two sea squirts ( *Didemnum candidum* ) belonging to Didemnidae, *S. clava* belonging to Styelidae and *Ascidia ahodori* belonging to Ascidiidae.

In this study, the complete mitochondrial genome information of YZ‐2024 was obtained, and the BLAST results confirmed its classification under class Ascidiacea, order Stolidobranchia, family Pyuridae, and genus Microcosmus. Its genome size and base composition ratios differ significantly from those of its congeneric species *M. sulcatus* , as well as from species in the same family such as *Pyura mirabilis* , *Herdmania momus* , and *Pyura gangelion*. The mitochondrial genome size of YZ‐2024 is shorter than that of *M. sulcatus* (15,028 bp; Gissi et al. 2010), *Pyura mirabilis* (15,778 bp; Kim 2023), and *Herdmania momus* (15,816 bp; Singh et al. 2009), and longer than that of *Pyura gangelion* (14,418 bp; Rubinstein et al. 2013). It is well known that the base composition of animal mitochondrial genomes exhibits a nonrandom pattern, and AT‐skew and GC‐skew values reflect biases in base composition (Sun, Sha, and Xiao 2021). The negative AT‐skew of YZ‐2024 indicates a preference for T; the base T and AT content is 47.16% and 74.0%, respectively. This asymmetrical base composition may be due to codon usage bias (Foster, Jermiin, and Hickey 1997; Singer and Hickey 2000), which further led to an asymmetry in the DNA strands (Liu et al. 2022; Wang et al. 2023). On the other hand, negative AT‐skew is possibly due to selective pressure during evolution that impacts mitochondrial DNA transcription or replication (Francino and Ochman 1997).

Gene rearrangement is an important manifestation of genome evolution (Tan et al. 2018), and the analysis of gene arrangement is an effective supplementary means to determine the taxonomic status and evolutionary degree of species (Hickerson and Cunningham 2000; Morrison et al. 2002). Aside from the COX1 gene, the gene arrangement sequence of the mitochondrial genome of YZ‐2024 differs from that of the other 22 species of Ascidiacea, which is a crucial basis for its significance in the analysis of ascidian evolution and development. Daric pionted out that ascidian genomes have been extensively rearranged in this fast‐evolving lineage (Daric et al. 2024). Rubinstein also found that each of the six ascidian mitochondrial genomes exhibited different and new gene sequences (Rubinstein et al. 2013). This evidence confirms the extreme plasticity of mitochondrial gene sequences in tunicates (Rubinstein et al. 2013). The result of the gene arrangement indicates that the species of Pyuridae are more primitive than those of Styelidae. Unexpectedly, YZ‐2024 and 12 species of Styelidae clustered into a clade, suggesting that YZ‐2024 is evolving in a more advanced direction. This also provides strong evidence for the polyphyletic evolution of Pyuridae. Compared to *M. sulcatus* , TRNA L and ND5 in YZ‐2024 translocated upstream, possibly as a result of environmental influences and evolution. In the genome of YZ‐2024, tRNA Pro and NAD4 are adjacent and overlap, and the distribution of the tRNA Pro‐NAD4 gene block covers more distant species on the phylogenetic tree. Therefore, it is speculated that this gene block was accidentally preserved or appeared in the genome of YZ‐2024 during frequent gene sequence rearrangements.

Sequence alignment analysis is an efficient and accurate method for species identification and classification, and phylogenetic trees can effectively illustrate the phylogenetic relationships among organisms (Pinheiro, Santander‐Jimenéz, and Ilic 2022). Evolutionary analysis indicates that YZ‐2024 is most closely related to *M. sulcatus* ; the genetic distance between them is 0.726. According to results of studies by Thorp, the genetic distance range between different populations of same species and different species of the same genus is 0.05–0.3 and 0.3–0.9, respectively; when the genetic distance between two species is greater than 0.9, they belong to different genera (Thorp 1982). Also, the phylogenetic tree produced in this study holds true to what is known about the evolution of the order Stolidobranchia; the phylogenetic positions of each species are consistent with the results of previous studies (Singh et al. 2009; Rubinstein et al. 2013; Wei et al. 2020). So, we determined that YZ‐2024 belongs to Microcosmus genus, family Pyuridae, order Stolidobranchia, class Ascidiacea. However, YZ‐2024 and *M. sulcatus* belong to Pyuridae, but they clustered with 12 species of Styelidae with high bootstrap value (> 90). This may be caused by gene rearrangement during the evolution of the species (Daric et al. 2024). The molecular characteristics above have provided evidence that YZ‐2024 is a new species of Ascidiacea. Additionally, we analyzed the PCGs of the mitochondrial genome of this species, enriching its molecular biology information and providing a reference for the identification and classification of future Ascidiacea species.

## 5 Conclusions

This study introduced a new species of sea squirt based on the morphological and molecular characteristics of the collected samples. This sea squirt has a smooth surface and lacks protuberances, with its tunica being orange‐red in color. It also lacks ganglia, and the tunica does not abruptly contract when startled. The heart of YZ‐2024 is oval‐shaped. The mitochondrial genome is 14,520 bp in size. Except for the COX1 gene, the arrangement of YZ‐2024 mitochondrial genome differs from that of other *Ascidiacea* species. YZ‐2024 is most closely related to *M. sulcatus* . The aforementioned features confirm that YZ‐2024 is a new species of sea squirt. Interestingly, YZ‐2024 belongs to Pyuridae, but it clustered with 12 species of Styelidae into a clade. Since there have been no reports on the molecular identification of sea squirts in the northern part of the South China Sea, this study represents the first. In future studies, in order to enrich the biological information of YZ‐2024, a search will be conducted for it in other sea areas of the South China Sea to determine its distribution range as well as its life history, reproductive habits, and identify the differences among the different sea squirts on the habitat types.

## Author Contributions

**Yichuan Zhang:** formal analysis (equal), software (equal), validation (equal), writing – original draft (equal). **Yuting Qin:** formal analysis (equal). **Yueying Wu:** conceptualization (equal), methodology (equal). **Liping Liu:** investigation (equal). **Wenguang Zhang:** resources (equal). **Ling Ding:** resources (equal). **Xiangpei Ya:** data curation (equal). **Zhiting Wen:** data curation (equal). **Kuaili Feng:** data curation (equal). **Hong Wang:** funding acquisition (equal), supervision (equal), visualization (equal). **Yujun Wang:** funding acquisition (equal), visualization (equal), writing – review and editing (equal).

## Ethics Statement

The experimental procedures of *Microcosmus* sp. z YZ‐2024 were conducted in accordance with the Guidelines for Care and Use of Laboratory Animals established by Beibu Gulf University, adhering to professional and academic standards as required.

## Consent

The authors have nothing to report.

## Conflicts of Interest

The authors declare no conflicts of interest.

## Data Availability

The sequence and annotation of *Microcosmus* sp. z YZ‐2024 mtDNA were submitted to the NCBI, with the accession number PP067884 in GenBank, which was released on January 22, 2024.
